# Novel botulinum neurotoxin-A tibial nerve perineural injection to alleviate overactive bladder symptoms in male rats

**DOI:** 10.1080/19768354.2022.2136239

**Published:** 2022-10-20

**Authors:** Seungbeom Kim, Hyun Seok Na, Jong Mok Park, Jin Wook Kim

**Affiliations:** a Department of Biomedical Science, Kyung Hee University, Seoul, Korea; b Department of Urology, Chungnam National University Hospital, Daejon, Korea; c Department of Urology, Chungnam National University Sejong Hospital, Sejong, Korea; d Department of Medical Informatics, Chung-Ang University, Seoul, Korea; e Department of Urology, Chung-Ang University Gwangmyeong Hospital, Gwangmyeong, Korea

**Keywords:** Overactive bladder, botulinum neurotoxin-A, tibial nerve perineural space, posterior tibial nerve, percutaneous tibial nerve stimulation

## Abstract

Although tibial nerve modulation has shown to induce positive changes in the overactive bladder (OAB), prolonged therapeutic effects using percutaneous stimulation have not yet been achieved. Intradetrusor onabotulinum toxin A injection can provide prolonged therapeutic effects; however, its delivery requires invasive measures. By applying local relief of tibial nerve neural entrapment with onabotulinum toxin A injection, this study investigated the feasibility and efficacy of combining the abovementioned two therapeutic strategies. An OAB animal model was developed using 12 adult Sprague–Dawley rats with cyclophosphamide intraperitoneal injection. A perineural injection site comparable to the tibial nerve perineural injection site and corresponding to that in humans was identified and developed in rats. The toxin was injected five days after establishing the OAB. The incision was made in the skin on the lateral surface of the thigh. The biceps femoris muscle was cut across, exposing the sciatic nerve and its three terminal branches: the sural, common peroneal, and tibial nerves, and 100 units of onabotulinum toxin A was injected into the surrounding tissue. Five days following injection, cystometry was performed. Inter-contraction time, contraction pressure, and interval of the disease state improved with statistical significance. The OAB animal model showed significant improvement with the tibial nerve perineural injection of botulinum toxin, thereby suggesting the possibility of a comparable treatment adaptation in humans.

## Introduction

Overactive bladder (OAB) is a very common age-related condition. Patients with OAB suffer from urinary urgency, usually accompanied with a manifestation of frequency and nocturia (Castro-Diaz et al. 2014; Eapen and Radomski 2016; Lightner et al. 2019; Kim et al. 2022). Approximately 11.8% of the US population has OAB. This number is expected to increase owing to an increase in the aging population. Consequently, the OAB represents a significant public health issue, which is exacerbated by the lack of any significant long-term curative options (Steers 2002; Stewart et al. 2003).

The previous primary treatment options, besides being unreliable and ill-documented, involved the use of anti-muscarinic agents. However, these agents have many severe side effects, including dry mouth, constipation, dyspepsia, and significant sluggishness. As a result, these treatment methods had high rates of drop-outs. One study reported that after 3 months, only <30% of patients still continued their treatment (Chughtai et al. 2008; Staskin et al. 2012). It is only recently that the use of beta-3 agonists has been proposed as a more sustainable treatment option. However, this treatment method also has its limitations. The primary starting dose of beta-3 agonists cannot be increased beyond a certain limit. In addition, they carry a risk of QT prolongation, which may cause sudden cardiac arrest (Steers 2002; Andersson 2017; Son et al. 2022).

Tertiary treatment methods are also limited because of the difficulty in implementation and unreliable outcome. As a result, no particular modality provides a stable treatment niche and the use of these treatment methods has steadily declined since their introduction (Cong et al. 2019; Hernández-Hernández et al. 2021). Sacral neuromodulation, once hailed as the quintessential treatment method for OAB as it taps directly into the theoretical neurogenic pathophysiology, is unreliable, prone to malfunction, and difficult to apply. Consequently, its practice is limited to only a select few tertiary centres (Birder and De Groat 2007; Castro-Diaz et al. 2014; Berthelot et al. 2019; Hernández-Hernández et al. 2021). A recent evolution of this line of treatment, percutaneous tibial nerve stimulation (PTNS), allows easier access to the nervous system through the peripheral route (Peters et al. 2010; Staskin et al. 2012; de Wall and Heesakkers 2017; Zeng et al. 2020; Girtner et al. 2021; Li et al. 2021). This route to the bladder seems, at first, unnecessarily circumventive. However, its proponents suggest that moving from behind the malleolus and into the sacral nerves via the tibial nerve can inhibit the sympathetic neurons at the level of Onuf’s nucleus. Although this does seem to afford short-term efficacy, it fails to maintain the prolonged effect beyond one week (Kim et al. 2020).

Intradetrusor onabotulinum toxin A injection is perhaps the most popular tertiary treatment option available so far. However, despite its efficacy, it still requires invasive cystoscopic intervention, as well as the use of significant amounts of a potent neurotoxin (Breuer et al. 2006; Chancellor et al. 2008; Baarini et al. 2021). Botulinum neurotoxin-A (BoNT-A) was introduced more than 40 years ago and is still widely used in the treatment of neuropathic pain and as a cosmetic. It binds to presynaptic cholinergic nerve terminals and decreases the release of acetylcholine (ACh). According to our current understanding, BoNT-A exhibits only transient effects that last several months, before ultimately disappearing. Hence, the current treatment paradigm dictates regular reinjection of BoNT-A (D’ancona et al. 2012; Jhang and Kuo 2018; Chen and Kuo 2020; Egeo et al. 2020).

However, several recent therapeutic strategies promise long-term efficacy, potentially by inducing atrophic anatomical changes in the pathophysiology of the target organs. Such a change can be brought about when BoNT-A acts indirectly on the target, in contrast to the direct effect where the inhibited release of ACh is first temporarily offset by axon arborization and then ultimately reversed by the return of functionality (Liao and Kuo 2015; Lin et al. 2019; Matak et al. 2019; Jiang et al. 2020; Kaya et al. 2020; Jung et al. 2021). By acting indirectly, the therapeutic strategy may circumvent this reversibility by inducing a long atrophic change to the target of the nerve, and not directly to the nerve itself (Welch et al. 2000; Waseem et al. 2011; D'ancona et al. 2012; Park and Park 2017; Salari et al. 2018).

An ideal strategy would be to utilize the ease of accessibility hinted by PTNS in combination with the relative long-term efficacy of BoNT-A. This may seem counterintuitive, because PTNS acts by eliciting neuronal activation, whereas BoNT-A is primarily a neuronal suppressor. However, we hypothesize that by facilitating an indirect approach using BoNT-A, not as a direct suppressor of neuronal activity, but to relieve the tibial nerve from the potential surrounding entrapment, we may indirectly affect the tibial nerve activation with an efficacy similar to that obtained using PTNS, thereby affording a long-lasting therapeutic window (Isner-Horobeti et al. 2015). The current study investigates this novel therapeutic approach in a rat model.

## Materials and methods

### Animals and research ethics

**Figure 1.**
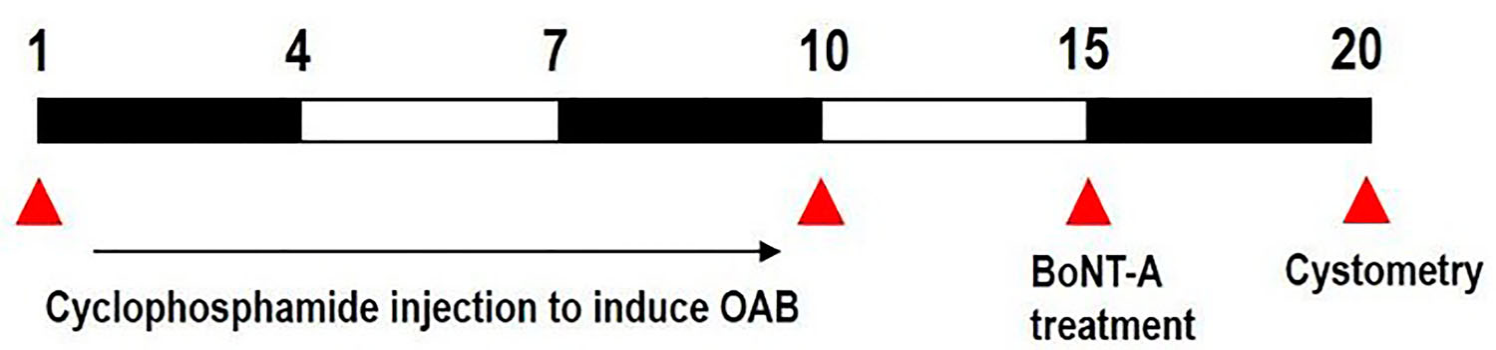
Experimental set-up involving development of cyclophosphamide-induced model, followed by BoNT-A injection and cystometry analysis.

We used 12 adult male Sprague–Dawley rats weighing 220 ± 5 g (10 weeks old), obtained from a commercial breeder (Orient bio Co., Sungman-Si, Korea). These experimental animals were classified into three groups (*n* = 4 in each group): the control group, the OAB-induced group, and the OAB-induced and BoNT-A-treated group (Figure 1). The experimental procedures were performed in accordance with the animal care guidelines of the National Institutes of Health (NHI) and were approved by the Institutional Animal Care and Use Committee (IACUC) of Kyung Hee University, Seoul, Korea [KHUASP(SE)-21-349].

### Induction of OAB

The rats of both the disease and treatment groups were administered with 75 mg/kg of cyclophosphamide through intraperitoneal injection (Sigma Chemical Co., St. Louis, MO, USA) every 3 days for 10 days to induce OAB symptoms, that is, urinary frequency and urinary incontinence. Next, this injection was stopped for 5 days for the treatment group before starting the BoNT-A treatment to maximize the effects of cyclophosphamide in the bladder of the rats.

### BoNT-A treatment

BoNT-A was administered 5 days after cyclophosphamide injection to fully induce the OAB symptoms. The rats were anesthetized with 10 mg/kg of Zoletil 50® (Vibac Laboratories, Carros, France) before BoNT-A administration. An incision was made in the skin on the lateral surface of the thigh and the biceps femoris muscle was cut across, exposing the sciatic nerve and its three terminal branches: the sural, common peroneal, and tibial nerves. Then, using a tuberculin syringe fitted with a 30-gauge needle, 100 units of BoNT-A (Allergan TM, Mayo, Ireland) were placed directly onto the exposed nerve and were allowed to flow onto the surrounding fascia to mimic the distributive behaviour of the toxin after injection at the target sites.

### Cystometry

Cystometry analysis was performed 5 days after the BoNT-A treatment to evaluate the improvement in urination elicited by BoNT-A. The bladder-contraction pressure, contraction time, and inter-contraction interval were evaluated 10 days after the OAB induction. After anaesthesia with intraperitoneal injection of 10 mg/kg Zoletil 50® (Vibac Laboratories, Carros, France), a transperitoneal incision was made and a polyethylene catheter (PE50) was inserted into the bladder. A syringe pump (Harvard Apparatus, Holliston, MA, USA) and a pressure transducer (Harvard Apparatus) were connected via a three-way stopcock to inject saline into the bladder and simultaneously record the intra-bladder pressure. After emptying the bladder, 0.5 mL of saline was infused, and the pressure-flow study was conducted. LabScribe (iWork System Inc., Dover, NH, USA) was used to measure the contraction pressure, contraction time, and inter-contraction interval of the bladder.

### Statistical analysis

Multiple comparisons were conducted by SPSS software (version 23.0, IBM Corporation, Armonk, NY, USA). Statistical analysis between the groups was conducted using one-way ANOVA followed by Duncan’s post-test. The data were presented as the mean standard error of the mean. *P* < .05 was considered statistically significant.

## Results

**Figure 2.**
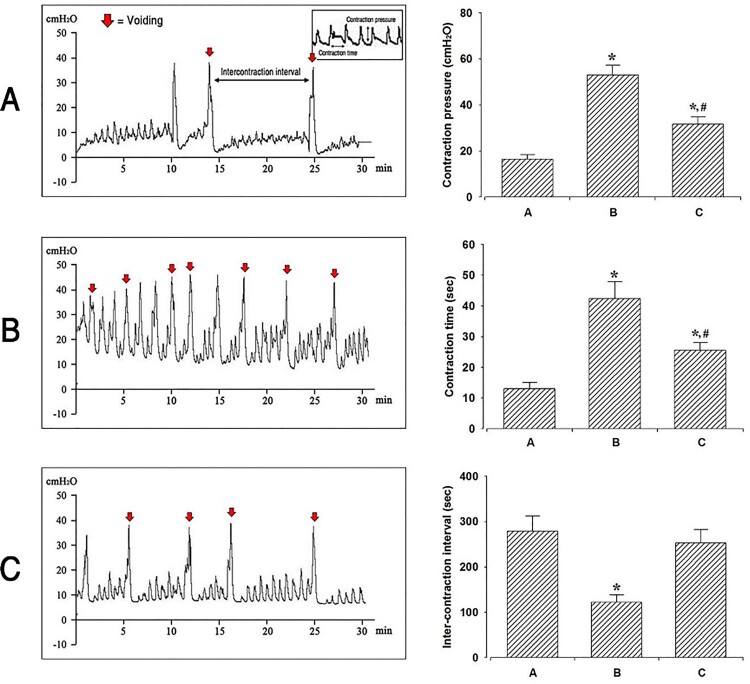
Effects of BoNT-A administration to the tibial nerve perineural space on bladder function through cystometry. Contraction pressure, contraction time, and inter-contraction interval amongst the three experimental groups: A: Control group; B: OAB group; C: Treatment group. *P < .05 compared to the control group. ^#^*P* < .05 compared to the OAB-induced group.

The cystometry analysis of the micturition function is presented in Figure 2. Induction of OAB with cyclophosphamide injection increased both the bladder-contraction pressure and bladder-contraction time and decreased the inter-contraction interval (graph B). These results showed that OAB was induced by repeated cyclophosphamide injections. Furthermore, these alterations in bladder factors ensued by the induction of OAB led to impaired voiding function (*P* < .05). However, BoNT-A injection into the perineural space of the tibial nerve decreased the OAB-induced contraction pressure and contraction time, but increased the inter-contraction interval (*P* < .05). These results indicate that micturition function, including bladder-contraction pressure, contraction time, and inter-contraction interval, which was impaired in the OAB-induced group, was improved by BoNT-A injection into the tibial nerve perineural space.

Groups A, B, and C are the control group, OAB-induced group, and OAB-induced and BoNT-A-treated group, respectively. The left panel indicates the recording of the bladder contractile pressure in each group, determined using cystometry. The right panel shows a comparison of the contraction pressure, contraction time, and inter-contraction interval amongst the three experimental groups. *P < .05 compared to the control group. ^#^*P* < .05 compared to the OAB-induced group.

## Discussion

Investigation of the locus of PTNS could provide an alternative explanation to the enhanced voiding control of perineural BoNT-A injection (de Wall and Heesakkers 2017). On the surface, the electromyogenic stimuli from PTNS seem to contradict the effects of BoNT-A injection because the PTNS involves constant stimulation, while the BoNT-A injection depresses the neural activity (Woo et al. 2020; Li et al. 2021). However, the anatomical structure of interest in our study is located on the posterior tibial nerve. In addition, previous studies have shown that the PTNS treatment can effectively modulate the bladder reflex pathways and the functionality of the lower urinary tract. Chang et al. (1998) demonstrated, through stimulation of the hind limb of rats, that electrostimulation of the posterior tibial nerve drastically decreases the C-Fos expression, which serves as a marker for metabolic activity. Peters et al. (2010) described the clinical data of outpatients with OAB and showed a decrease of 20.325% in the mean voiding frequency after weekly administration of PTNS for 12 weeks. They also showed that the PTNS treatment does not show long-term effects. Based on the abovementioned clinical evidence on the efficacy of the neural circuit that the PTNS utilizes, we could locate the site of injection for BoNT-A and actuate a neural pathway identical to that utilized for PTNS by using the surrounding fascia (layer of tissues) of the posterior tibial nerve as the site of injection (e.g. the tarsal tunnel). Thus, it is imperative to investigate how the anatomic structure of the tarsal tunnel impacts the stimulation of the posterior tibial nerve via tarsal tunnel syndrome and how perineural injection sites can actuate the neural pathway of PTNS (McSweeney and Cichero 2015; Kiel and Kaiser 2018).

The tarsal tunnel syndrome, or the distal tarsal tunnel syndrome (DTTS), is an entrapment syndrome of the posterior tibial nerve, first described by Keck (1962). It is a focal compressive neuropathy involving the tunnel deep in fascia, the flexor retinaculum, and within the abductor hallucis muscle of the foot/ankle, leading to a range of symptoms affecting the plantar margins of the foot as it compresses the posterior tibial nerve that bifurcates inside the tarsal tunnel (Antoniadis and Scheglmann 2008; Baarini et al. 2021). Compression of the posterior tibial nerve causes its dysfunction and extends the spectrum of symptoms to the lower urinary tract, because an anatomical deformity results into compressive stimulus on the nerve (McSweeney and Cichero 2015; Baarini et al. 2021). To further elucidate the notion of tarsal tunnel influencing the bladder behaviour, stimulation of an area of the nervous structure, that is posterior tibial nerve running from the posteromedial lower leg region to the planter region, appears to modify the behaviour of the innervation of another nervous structure, which is part of the innervation system in the bladder, thus altering the bladder behaviour. Pudendal and pelvic nerves are such nervous structures that affect the behaviour of the bladder. Numerous studies have confirmed the efficacy of BoNT-A administration for the treatment of the entrapment syndrome, specifically the release of functional compression of the nerve structure (Breuer et al. 2006; Tsai et al. 2006; Antoniadis and Scheglmann 2008; Law et al. 2010; Isner-Horobeti et al. 2015; Juan-García et al. 2016; Baarini et al. 2021). Considering that BoNT-A has been shown to be efficient in the treatment of several other entrapment syndromes through the release of functional compression, we could derive a therapeutic effect on OAB-induced lower urinary tract dysfunction with the release of neural entrapment. BoNT-A injection helps release the functional compression, that is the entrapment, to the surrounding fascia or muscle, thereby releasing tension and improving the neural conditions and correcting the defects related to bladder functionality. Alternatively, it can also explain how the stimulatory impact of the posterior tibial nerve by botulinum toxin would transfer to the other innervation system, the pudendal and pelvic nerves, and improve the neural dysfunction of the bladder.

PTNS is based on the acupuncture techniques of the traditional Chinese medicine and does not have a clear basis in modern medicine (Chang et al. 1998; Wu et al. 2016). Although DTTS is presented as a compression syndrome involving a specific anatomical pathology, no clear association has alluded to the mechanism of PTNS. Zeng et al. (2020) demonstrated in rodents that electrical stimulation of the tibial nerve not only affects the anal canal and vaginal mucosae through motor output from the spinal cord, but also sends the signals up to the pontine micturition centre (PMC) and the cerebral cortex, which are neurological structures that manage the secretion of the motor neurotransmitter acetylcholine (ACh) in the lower urinary tract. Although electrical stimulation by PTNS could depress the excessive release of acetylcholine from the pelvic and pudendal nerves, the release of the posterior tibial nerve in DTTS via BoNT-A injection could also achieve a similar effect by alleviating constant stimulation on the posterior tibial nerve caused by nerve compression.

The long-lasting effect of BoNT-A compared to the electrical stimulation of PTNS is another significant advance of the OAB therapy that differentiates it from other tertiary treatment methods. The mean duration of the efficacy of BoNT-A is 7–8 months in contrast to the short-lived positives achieved by electromyogenic stimulation with the PTNS, which lasts for days or weeks at most (Kaya et al. 2020; Zeng et al. 2020).

**Figure 3.**
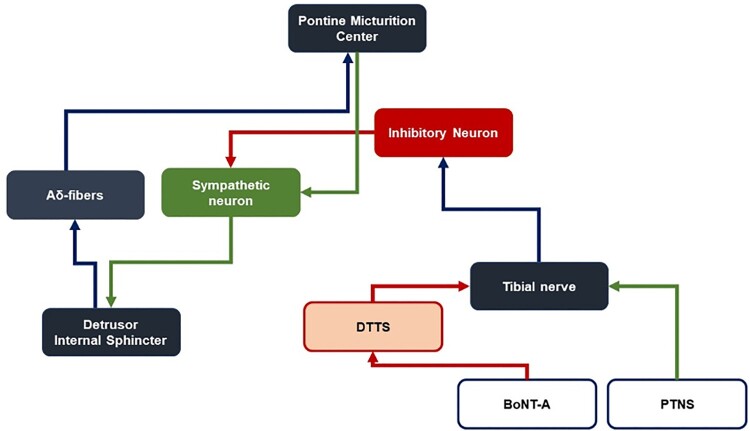
Schematic comparison between the treatment mechanism of the current study and that suggested by DTTS and conventional PTNS.

Systemic effects could be induced through the action of BoNT-A on Substance P and subsequently on nociceptive A-delta fibres (Chien et al. 2003; Matak et al. 2017). The ensuing absence of afferent feedback on the PMC leads to deflated descending efferent signals to the detrusor muscle and internal sphincter, both of which are under involuntary control. Alternatively, BoNT-A conjugates the same neural pathway as PTNS that triggers inhibitory neurons to act on sympathetic neurons. However, BoNT-A may act specifically and indirectly on posterior tibial nerves within the distal tarsal tunnel, releasing pressure on the tibial nerves and thus allowing a similar but more long-lasting effect compared to that achieved through PTNS (Figure 3).

The perineural space of the posterior tibial nerve of rats is structurally different from that of the human: the tibial nerve of the rat bifurcates at the fibula region of the hind limbs, whereas that of the human bifurcates into lateral and medial branches at the tarsal tunnel – an anatomical structure that rodents do not have (Liu et al. 2018). The OAB symptoms were, however surprisingly, alleviated after BoNT-A perineural injection, implicating the presence of alternative comparable pathways. Using biological markers for botulinum toxin, including C-fos and SNAP-25 cleavage, several previous studies have attempted to confirm the routes for the movement of the toxin (Nugent et al. 2018; Mechaly et al. 2021, 2022). Specifically, evaluating the expression of SNAP-25 on the ventral root of nerve roots L4–S4 may provide clarity to determine the efficacy of the distal injection site.

Another important concern regarding the novel injection site is the potential adverse effects on the local musculature, particularly temporal paralysis, caused by passive diffusion of the agent (Peters et al. 2010; Staskin et al. 2012; Berthelot et al. 2019). The kinetic function of the rats of the treatment group appeared normal. Hence, it is another highly inefficient measure to ensure the normal kinetic function in human patients after the treatment. Although the safety device designed specifically for the injection is expected to enhance the accuracy of the injection and thus limit the undesirable diffusion of the toxin, additional studies should be conducted to explain the migration and diffusion of BoNT-A from the site.

Further examination of the effects of nerve compression, particularly evaluation of the acetylcholine activity at the affected presynaptic ends on distant nerves, to determine whether the anatomical deformity leads to excessive stimulation of distant organs and subsequent dysfunction of the structure will be highly useful to increase our current understanding of the topic. However, the spectrum of the research on physiological diseases is extremely wide because of the numerous anatomical and physiological factors involved in nerve compression, such as inflammation, compression angle, and physical damages on the nerve, which blind the clinical diagnosis. The inflammation, compression angle, and physical damages on the nerve will affect the neurotransmission that extends to the innervation of distal musculature, which determine how the anatomical deformities affect the nerve. Thus, distant innervated organs may play a role in the diagnosis and subsequent treatment of the condition.

## Conclusion

This study demonstrated an improvement in the voiding function through the BoNT-A tibial nerve perineural injection in an OAB rat model. BoNT-A suppressed the bladder-contraction pressure, reduced the bladder-contraction time, and increased the inter-contractile interval, indicating diminished urination frequency. Research into new therapeutic approaches to maximize the therapeutic efficacy or minimize the side effects can provide opportunities for developing novel treatments.

Our study has several limitations also. Inflammation or pain is a critical feature in assessing the depression of afferent signals in the bladder because it is one of the primary symptoms observed in OAB patients. However, this factor was not measured in this study. Furthermore, the perineural space of the posterior tibial nerve in rats is not structurally identical to that of humans. Hence, the results achieved using rats cannot be extended to human patients. The neural circuit that the pharmacological agent takes in reaching the bladder is obscured. Hence, it is still too soon to say that this treatment method is a sufficient alternative to the current state of clinical interventions. These limitations necessitate further investigations at the molecular scale to substantiate the neural route of BoNT-A in modifying the bladder function and to evaluate the inflammatory changes in the bladder tissue following the treatment.

Nevertheless, the study is a successful preliminary investigation into a promising novel treatment method, which may be used to develop a long-term tertiary treatment method for treating unresolved OAB cases.
